# What is the next step of ICT development? The changes of ICT use in promoting elderly healthcare access: A systematic literature review

**DOI:** 10.1016/j.heliyon.2024.e25197

**Published:** 2024-02-07

**Authors:** Sihui Chen, Mengyuan Niu, Cindy Sing Bik Ngai

**Affiliations:** a The Department of Chinese and Bilingual Studies, The Hong Kong Polytechnic University, Hong Kong; b Division of Public Policy, The Hong Kong University of Science and Technology, Hong Kong

**Keywords:** Information and communication technology, Elderly, Healthcare, COVID-19

## Abstract

The objective of this study was to undertake a comprehensive review of the evidence published, with a focus on understanding the experiences of the elderly in leveraging Information and Communication Technology (ICT) for their healthcare needs during the COVID-19 period. In compliance with the Preferred Reporting Items for Systematic Reviews and Meta-Analyses (PRISMA) guidelines, this review scrutinized all peer-reviewed articles in English sourced from PubMed, PsycINFO, Scopus, and Web of Science, targeting studies that focused exclusively on the elderly within the COVID-19 timeframe, incorporated ICT-based technology as intervention, and were associated with the assessment of the process of employing ICT for healthcare needs. The search strategy identified 1752 records, of which 34 studies met the inclusion criteria. The functionality of ICT was categorized, types of barriers were identified, and the subsequent changes that the elderly population underwent were synthesized and deliberated. This review offers valuable insights into the elderly's subjective experiences in utilizing ICT, which may offer guidance for future ICT development geared towards enhancing the well-being of the elderly. Future research should incorporate the perspectives of relevant healthcare providers in evaluating the effectiveness of ICT usage. Further studies are also needed on underserved elderly groups to provide a more holistic view.

## 1 Introduction

The development of Information and Communication Technology (ICT) has enabled the provision of remote-distance healthcare services. ICT encompasses various technologies such as mobile apps, network equipment, and software applications to provide services like videoconferencing and e-learning to facilitate healthcare information exchange and support [1,2]. ICT has expanded to the healthcare sphere, facilitating high-quality and effective healthcare-related products and services, including electronic health (e-health), mobile health (m-health), telemedicine, telehealth, wearable technology, assistive technology, and electronic health records (EHRs) [3]. With the emergence of the COVID-19 pandemic, the adoption of ICT has further accelerated [4].

Due to social isolation restrictions during the COVID-19 pandemic, hospitals, physicians, nurses, and other healthcare providers adopted ICT at a faster pace to overcome geographical constraints to meet patients’ demands [5,6]. As of 2023, the U.S. region accounted for nearly 36 percent of the global ICT market share, followed by the EU and China, which ranked the second and third biggest regions, each accounting for a market share of over 11 percent [7]. Recent studies have shown that the elderly are increasingly using ICT to meet their daily needs such as shopping, socializing, and entertainment, compared to the pre-pandemic times [8]. The elderly have shown a sustained interest in utilizing ICT to support their healthcare, wellness, and communication needs even in current times.

The use of ICT during the pandemic has been widely acknowledged as beneficial for enabling the elderly to maintain social connectedness, access information, and receive healthcare services [[8], [9], [10], [11], [12]]. However, it is important to note the challenges posed by age-related physiological and biomechanical changes that may impede the adoption and use of ICT by the older population [[13], [14], [15], [16]]. These include a lack of digital literacy, insufficient access to technologies and devices and other obstacles [9,11]. Moreover, questions remain about whether ICT has been adequately adopted to improve healthcare services for the elderly.

Before the COVID-19 outbreak, studies on ICT in healthcare had primarily focused on institutional problems such as attempting digital transformation for a large-scale healthcare implementation [17,18]. Other studies have focused on the outcomes of using ICT such as quality of life [5,[19], [20], [21]] and changes in mental well-being [9,22,23]. Discussions of such topics are insufficient to shed light on the experiences of ICT use by the elderly during the pandemic. To address this gap, a more nuanced examination of ICT use from the elderly's perspective and an analysis of changes during the pandemic are needed.

Thus, our study aimed to conduct a systematic literature review to fill the gap in research on aging-population-based ICT use and to provide an in-depth understanding of the elderly's subjective experiences in utilizing ICT throughout the COVID-19 period. Specifically, we investigated the complete process of using ICT, including the types of ICT that have been widely adopted, the perceived barriers faced by the older population, and the changes that occurred after using ICT. To guide our investigation, the following research questions were asked:

(1) What functions of ICT were addressed in elderly healthcare during COVID-19?
(2) What were the barriers to using these types of ICT during COVID-19?
(3) What were the substantial changes after using ICT during COVID-19?

The following sections of this paper provide a comprehensive overview of the research process and findings. The Materials and Methods section provides a detailed account of the literature search and review process employed to identify relevant studies for analysis. The Results section presents the research findings, emphasizing the significant barriers and changes observed regarding the use of ICT tools for healthcare among the elderly. Lastly, the Discussion section explores the implications of these findings and their potential influence on the design and implementation of ICT-enabled health services for the elderly in the post-pandemic world, as well as the limitations of this study.

## 2 Materials and methods

This study employed the Preferred Reporting Items for Systematic Reviews and Meta-Analyses (PRISMA) methodology to conduct this review. PRISMA was developed to enhance the quality of systematic reviews through a rigorous checklist covering aspects such as the objectives, methods, and eligibility criteria [24]. The checklist items have since been further refined based on recommendations to include additional details, such as fully reporting on the search strategies for all databases rather than just one and describing any automation tools used during the various stages of the review (e.g., the study selection process), as well as the procedures for determining study eligibility for each synthesis [25]. In adherence to the PRISMA methodology, this study was not formally registered but provided a clear account of the review process covering the PRISMA checklist items, which can be found in the supplementary materials at the end of the paper.

### 2.1 Search strategy and information source

This research study employed the use of ICT as an intervention to explore the experiences of the elderly population in procuring healthcare resources during the COVID-19 pandemic. In order to pinpoint pertinent studies, we employed the PICO (Patient/Population, Intervention, Comparison, Outcomes) framework, an established approach in systematic health intervention reviews, to facilitate the search for suitable studies and the synthesis of characteristics necessary for interpreting results beyond a meta-analysis [26].

In line with the PICO framework, we delineated the scope of the review and sourced matching studies from a range of databases, including PubMed, PsycINFO, Scopus, and Web of Science. The population of interest for this study (P) was the geriatric demographic, specifically individuals aged 50 years and above, who had the chance to employ ICT for healthcare purposes amid the COVID-19 pandemic. The intervention (I) encompassed the use of any ICT variant. The comparison group (C) compared types of elderly if two or more groups received different interventions. Although the main focus of this study was to understand the overall experiences of the elderly population, not all PICO aspects were necessary [27]. This study did not involve a comparison aspect. The outcome (O) of this study was to measure the process of elderly individuals utilizing ICT to access healthcare services.

Consequently, we determined the search keywords (see Table 1) to locate relevant studies. Considering the broad definition of ICT, our search terms also included terms associated with the application of digital technologies to healthcare such as e-health, m-health, telemedicine, telehealth, and other analogous terms, to ensure a comprehensive coverage in our review.

**Table 1:** Key search terms.

Population-related Keywords	Intervention-related Keywords	Outcome-related Keywords
“Elderly” OR “Older Adults” OR “Seniors” OR “Retired people” OR “Elders” OR “Aged people” OR “Aging” OR “Old age”	“Information and Communication Technology” OR “ICT” OR “e-health” OR “m-health” OR “Telemedicine” OR “Telehealth” OR “Wearable technology” OR “Assistive technology” OR “Electronic health records (EHRs)”	Healthcare* OR “Medical treatment”

### 2.2 Inclusion and exclusion criteria

In accordance with our research questions, the initial inquiry covered our established criteria within the context of the “COVID-19 period", “ICT-related interventions", and the demographic of the “elderly" population. The subsequent two research questions focused on examining the barriers and changes encountered during or subsequent to the adoption of ICT. The inclusion of studies in this review was therefore contingent upon their ability to adequately address all three research questions. Table 2, Table 3 contain comprehensive information regarding the screening criteria in each step.

**Table 2:** Screening of articles by title and abstract (step one).

Inclusion Criteria	Exclusion Criteria
Empirical article	Literature review article
Within the required research date COVID-19 period test	Out of the required research date Not COVID-19 related
Only target on elderly	Include more population groups
Include ICT-related technology as the intervention	Not ICT-related intervention
Associated with evaluating the process of using ICT for healthcare	Not associated with evaluating on the use of ICT for healthcare

**Table 3:** Screening of articles by full text (step two).

Inclusion Criteria	Exclusion Criteria
Full-text available	Full-text not available
English version	English not available
COVID-19 period test	Not COVID-19 related
Only target on elderly	Include more population groups
Include ICT-related technology as the intervention	Not ICT-related intervention
Associated with evaluating the process of using ICT for healthcare	Not associated with evaluating on the use of ICT for healthcare

To guarantee the review's rigor, several criteria were met. First, included studies were those conducted between 2020 and 2022, aligning with the COVID-19 pandemic chronology. Second, only English publications were included and reviewed. Third, articles had to be empirical studies retrieved from the database searches. Finally, the articles focused exclusively on the elderly population instead of multiple age groups.

### 2.3 Data selection process

Five key steps were taken in this study. Initially, 1752 records were retrieved from four electronic databases: PubMed (n = 632), PsycINFO (n = 42), Scopus (n = 554), and Web of Science (n = 524). After importing them into EndNote, 436 duplicates were automatically removed, lowering the total to 1316 records. Uploading them to Rayyan, an open-source web application for systematic reviews [28] enabled two reviewers to identify additional duplicates manually using similarity checks of above 90%, yielding 1177 records. Next, 24 records lacking abstracts were excluded prior to title/abstract screening as per selection criteria, leaving 1129 records.

**Fig. 1:**
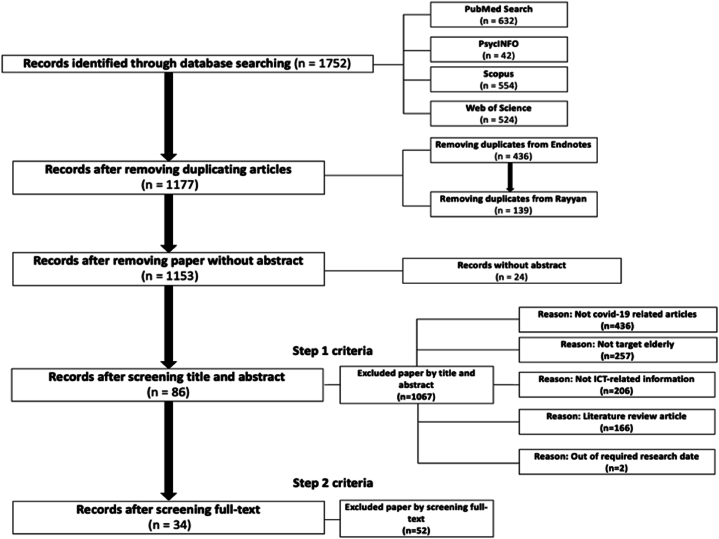
Flowchart detailing inclusion/exclusion process.

After the review of titles and abstracts, both reviewers decided to eliminate 1067 papers due to various reasons. These included a lack of relevance to COVID-19 (n = 436), not being focused on the elderly group (n = 257), not related to ICT information (n = 206), being literature review articles (n = 166), and not falling within the required research time frame (n = 2). This left a total of 86 studies for further consideration. Within these 86 studies, some studies indicated high relevance for inclusion criteria but were finally excluded for being focused on beyond the elderly group [[29], [30], [31]], and their lack of association with the evaluation of the ICT use process [[32], [33], [34]]. Finally, full-text review by both reviewers resulted in the inclusion of 34 papers in this analysis (See Fig. 1).

## 3 Results

### 3.1 Functions of ICT

**Fig. 2:**
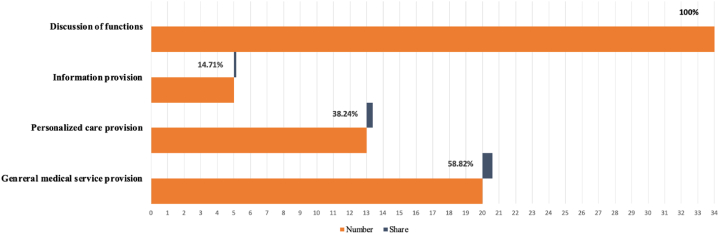
Categorization of functions of ICT from included studies.

Of the selected articles (See Appendix A in supplementary materials for full characteristics), this study aimed to address the first research question regarding which functions of ICT were addressed in elderly healthcare during COVID-19. The types of ICT in healthcare were diversified and multifunctional because such technologies could be derived into different products. Through mobile and software applications, the literature demonstrates ICT facilitated telehealth [[35], [36], [37], [38]], telemedicine [[39], [40], [41]], teleconference [[42], [43], [44]], and related modalities. To synthesize how the selected studies deployed ICT to deliver healthcare during the pandemic, this study categorized (see Fig. 2) the technologies into three major functional classifications: general medical service provision (n = 13), personalized care provision (n = 20), and information provision (n = 5), accounting for 38%, 59%, and 15% of the literature respectively.

#### 3.1.1 General medical service provision

One of the main functions of this kind of ICT is to provide a variety of services for the elderly, especially general medical services for the instrumental activities of elderly daily living. The general medical services identified by the selected studies included regular communication with healthcare providers [45], professional neuropsychological evaluation [43], real-time access to healthcare resources [46], and cognitive training programs [47]. Additionally, due to the limited opportunities for caregivers to participate in in-person coping mechanisms as well as socialization and support during the pandemic, developed countries facing aging challenges such as the United States and Canada made efforts to expand and diversify home and community-based services. The scope of medical service provision extended to include additional ancillary services intended to circumvent social isolation and foster health information through online healthcare-related activities. Exemplars included virtual forums addressing the elderly living with dementia [44,48], vaccination consultation sessions [38], and health promotional programs [[49], [50], [51]].

#### 3.1.2 Personalized care provision

This included studies categorizing ICT applications supporting personalized elderly care into three areas: care for elderly patients with pre-existing medical conditions (n = 16), assisting with wearable and mobile technologies (n = 3), and daily living assistance (n = 1). Each of these areas constituted 80%, 15%, and 5% respectively of the papers related to personalized care provision.

Firstly, ICT facilitated personalized care for elderly patients with pre-existing medical conditions. Due to pandemic-imposed social restrictions, physicians were compelled to transfer the regular treatment of patients to an online mode. Therefore, studies documented ICT-enabled video therapy and teleconsultations supporting Parkinson's patients [52,53], dementia [39], HIV [54], chronic and/or treatment-resistant affective disorders and comorbid personality [55], diabetics [41,56] and cancer [57,58]. In addition, personalized care delivered via ICT allowed medication reconciliations to check for changes and needs [38], as well as rehabilitation aiming to improve physical outcomes and reduce falls [52].

The second area of personalized care was about wearable or mobile technologies. These technologies primarily function to facilitate monitoring [60,61], in addition to providing support and assistance with health and cognition [59,61]. With these digital records, healthcare providers were able to note and ensure intensive service provision. For instance, wearable technologies embedded in smartwatches and fitness trackers were adopted so that elderly patients who were experiencing comorbidities could utilize such technology for monitoring to ensure their optimal health [60]. The elderly could also utilize a telemonitoring app to receive integrated healthcare services from general practitioners and care coordination centers [60].

The third area of personalized care was to provide assisted services for elderly daily living. The elderly could make shopping or food requests easily by calling the relevant department [38]. Instead of functioning as a primary service within the online service platform, this daily living service operated as a supplementary provision to enhance the overall care system during the COVID-19 period. Consequently, offline services could be specifically directed towards the treatment of COVID-19-related illnesses.

#### 3.1.3 Information provision

Five studies addressed the function of information provision. Though information provision did not constitute a direct form of care, it still played a valuable role during the COVID-19 period. In China, people aged over 40 treated short phone messages more seriously than people aged between 18 and 25 [62]. The messages contained timely information related to anti-epidemic alerts and suggested behaviors to protect their health. In a virtual health ministry project conducted in one African American religious community in the United States, the dissemination of information through Facebook and Instagram became a major step in encouraging the elderly to join the virtual healthcare provision scheme [42]. Through phone calls to the elderly, vaccination information and available appointment dates were conveyed to the elderly for their records [38]. The computer or cell phone also played the role of a media, which enabled the elderly to get more information through communicating with their friends, attending online activities, online shopping, etc. [63,64].

### 3.2 Barriers in using ICT

**Fig. 3:**
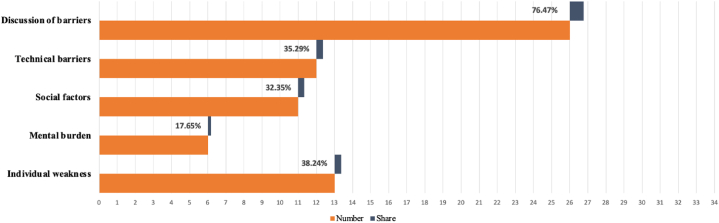
Categorization of barriers using ICT from included studies.

Another research aim was to ascertain the barriers to utilizing ICT from the elderly's perspective during the COVID-19 pandemic. There were 26 out of 34 articles (76%) which examined the barriers faced by the elderly regarding ICT-related healthcare (See Fig. 3). To comprehensively understand these challenges, this review categorized the barriers that the elderly were facing into four parts: individual weaknesses (n = 13, 38%), mental burden (n = 6, 17.6%), social barriers (n = 11, 32%), and technical barriers (n = 12, 35%).

#### 3.2.1 Individual weaknesses

Among the 26 included studies, 13 papers (50%) addressed the weaknesses of the elderly in using ICT-assisted healthcare, which extended beyond their socio-demographic disadvantages, health status, digital literacy, and previous experiences accessing ICT - all of which constituted facets of individual challenges in ICT usage. Among the socio-demographic disadvantages, age was the first challenge identified from their socio-demographic records. Age hindered the elderly's interest in adopting healthcare wearable devices [60]. Decreasing interest in adopting or learning about new technology followed next by eliminating greater possibilities in accessing telemedicine [37,41,64]. Apart from age, there were other social demographic factors that were spotted impeding the use of ICT in healthcare, such as lower income [37,64,65] which limited the elderly's capacity of purchasing ICT devices. Lower education levels, language barriers, and gender disparities all contributed to individual weaknesses and led to more dissatisfying outcomes in a telehealth visit [41,54,56,64].

Lack of experience in using ICT was one essential weakness preventing the elderly from better access to ICT-assisted healthcare. The identified papers highlighted the impacts of experience shortfalls in elderly access to telemedicine. Witt et al. [53] indicated that 84.6% of their investigated Parkinson's patients had no experience in trying telemedicine. The lack of previous experience in participating in teleconferences or handling technique tools was unhelpful for the elderly to switch from an offline to online consultation mode during the COVID-19 period [37,66].

Limited digital literacy posed another challenge. Although patients could use email to resolve dementia-related behavior disorders, the lack of digital literacy was still a concern for equal access to email approach [39]. Due to the lack of digital literacy, the virtual delivery of deprescribing services [65] and telehealth over video platforms [39] faced obstacles during the COVID-19 pandemic.

The health status of the elderly was another challenge when attempting to utilize ICT for healthcare. Physical health and cognitive health were relevant in this regard. Due to functional impairments [45,66], particularly in hearing and vision [58], the elderly experienced difficulties accessing computers and the Internet to have video or phone calls with health providers. Studies also identified that self-reported poor health reduced the elderly's willingness to attempt to use wearable and mobile technologies [61], as well as the motivation to learn new technology to participate in online activities [64]. Additionally, cognitive health issues caused short-term memory loss when they were using home-based telemonitoring apps [59]. Cognitive impairment also resulted in difficulty comprehending interview questions during telemedicine [40].

#### 3.2.2 Mental burden

Mental stress was a significant barrier for the elderly in using ICT. Anxiety is first identified when encountering new technologies or telemonitoring apps [43,59]. Lack of confidence in participating in home telehealth visits [67], perceived low quality of care [41], and lower acceptance of adopting a cognitive training program [48] further exacerbated the mental burden and impeded the possibility of choosing ICT to provide healthcare. Furthermore, the elderly expressed privacy concerns regarding wearable and mobile technologies and telemedicine as these technologies involved the transformation and handling of sensitive information [43,61]. When transmitting information via telemedicine, the elderly exhibited a reluctance to comply with vaccine instructions during telemedicine sessions, indicating a lack of trust [38].

#### 3.2.3 Social barriers

In addition to the barriers caused by individual discrepancies and mental burden, various social factors affected the elderly's behavior and attitudes towards the use of ICT. The first social barrier identified was the lack of interpersonal contact during the pandemic period, which led to misinformation between clinical and non-clinical staff [38]. The absence of face-to-face contact has been found to hinder patient-clinician connection [57]. Telemedicine has been considered inferior to in-person care as a result of lacking eye contact and hands-on physical diagnosis [39,57]. Lack of offline consultation when conducting deprescribing could result in a greater propensity for withdrawal effects and a higher risk of hospitalization [65].

The second social barrier was the lack of adequate resources to facilitate access to ICT access for the elderly. This included both material and human resources. While some elderly individuals had smart electronic devices, they did not have equal access to video-conferencing or virtual care [65,66]. In Greece, the presence of numerous isolated areas in island and mountain villages shows the importance of having essential technological equipment in order to effectively utilize telemedicine services [43]. The absence of volunteers and participants, as well as the intensity of time and resources required, hindered engagement in technology training programs aimed at alleviating social isolation and loneliness [63]. Lack of social support and family members’ assistance made it difficult for the elderly to access telehealth services independently [42,56,65]. They relied on trusted individuals and preferred to ask them for help when the Internet was inaccessible [44]. If there was no perceived support from a trusted person, ICT access was impossible.

Other social barriers included trust in affiliated religious communities [42] and having private or public insurance [41]. African- Americans were more likely to trust their community and turn to telehealth for clinics than other ethnicities during the COVID-19 pandemic, due to their faith-based model [42]. Elderly patients without private insurance for telemedicine were less willing to try such technologies [41].

#### 3.2.4 Technical barriers

Technical challenges represented a significant barrier to engaging the elderly in using ICT for their healthcare. Internet inaccessibility was one such challenge that hindered access to ICT and reduced the quality-of-service delivery through video telehealth [36,44,46,54,56,67]. A community in Bangladesh that aimed to serve aged people with diabetes found that a bad Internet signal was an obstacle for the elderly to access telehealth services [56]. The accessibility of the Internet and technological devices was a key parameter for benefiting from telehealth services, as indicated by a telephone survey of elderly individuals aged over 65 [37]. Unstable networks in rural areas decreased the participation rate in video, voice calls, and conferences, further limiting access to telehealth services [36,44].

Technical problems with the device or programming design were also a concern. Virtual programming limitations could distract older adults with Alzheimer's and dementia from attending virtual sessions [48]. Weighing non-verbal expressions on the computer screen made it difficult for the elderly to cooperate with advanced care planning using video conferencing [44]. The interface design was criticized as unfriendly for older users in some smartphone-based healthcare apps [68]. Technical issues could also be encountered during the process of telerehabilitation, where participants had difficulties understanding the procedure the first time [52].

### 3.3 Changes after using ICT

**Fig. 4:**
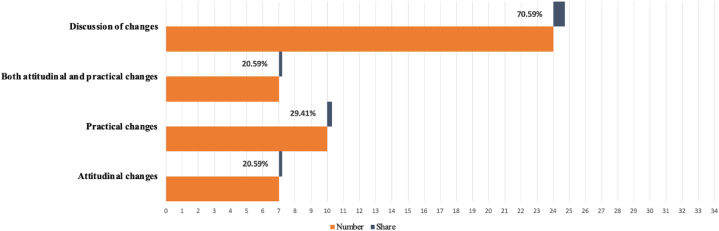
Categorization of changes from using ICT from included studies.

The last research question of this study aimed to address the substantial changes after using ICT during COVID-19. Li et al. [64] found that elderly individuals who did not use ICT were more likely to experience social isolation, higher levels of loneliness, and cognitive decline compared to ICT users. This indicated that observing changes in older adults after adopting ICT might provide some valuable insights into the future development of ICT and enhance elderly healthcare efficiency. 24 out of 34 articles (n = 24, 70%) observed changes among older adults after adopting ICT during the COVID-19 pandemic (See Fig. 4). Specifically, the changes after ICT use could be categorized into two types: attitudinal changes (n = 7, 20%) and practical changes (n = 10, 29%). Monitoring both attitudinal changes as well as practical changes (n = 7, 20%) could provide a comprehensive view of how ICT impacts the elderly.

#### 3.3.1 Attitudinal changes

The reviewed literature highlighted shifts in the attitudes of elderly patients toward ICT. In Bangladesh, with the service engagement from the living community, there was a surge in openness towards ICT among the aged particularly those with antecedent exposure to telehealth service delivery [56]. The provision of technical assistance was perceived by German seniors with Parkinson's as crucial for the successful deployment of video therapy modalities [53]. In addition, a video intervention for the elderly with mild dementia in Canada expressed full acceptability towards video telehealth services [44]. Elderly patients extensively commended the quality of health-related information conveyed via text messages during the COVID-19 crisis, and thus acknowledged the significance of this communication mode [62].

Older adults indicated a preference for video telehealth, asserting that clinicians could deliver more personalized care through this medium [57]. By transmitting non-verbal cues, such as body language, video telehealth enabled older patients to perceive a heightened level of empathy, understanding, and connection from clinicians. Within the context of online group sessions, the elderly perceived a sense of validation as these sessions provided comprehensive feedback on their tasks, alongside succinct weekly individual engagements with therapists [55]. The endeavors of group facilitators were applauded, and the elderly participants reported an enhanced sense of belonging associated with the virtual support [48].

#### 3.3.2 Practical changes

Of the selected articles, the elderly participants noted the efficacy of using video telehealth services as an accurate alternative to conventional pen-and-paper methods for assessing patients' neuropsychological functioning [43]. Conserving traditional clinical space and time was frequently cited as a benefit by the elderly participants [38,39]. In Turkey, existing dementia-related issues could be addressed before the elderly visited the hospital during its peak times [39]. Patients requiring emergent care could be directed to relevant departments or emergent services centers accordingly and effectively [39]. Furthermore, the shift towards telemedicine freed up space to establish a COVID-19 assessment center during the pandemic [38]. Studies reported a substantial increase in the utilization of telehealth services by the elderly during the outbreak in the United States [64,69]. This increase led to an increase in computer time and physical activity participation, while transportation time to hospitals decreased [50].

In addition, advancements in telerehabilitation contributed to the enhancement of walking performance in elderly individuals experiencing Parkinson's disease-related symptoms [52]. These patients were guided in executing daily living and mobility tasks, leading to higher quality-of-life scores post-intervention. The utilization of ICT was also linked to improved mental health among older adults [49,51]. Specifically, telehealth-delivered psychological training courses were associated with reduced stress and loneliness, as well as increased resilience, happiness, wisdom components, and positive perceptions of aging [49].

Furthermore, the use of ICT was observed to significantly decrease the stress levels of caregivers for elderly individuals [51]. However, the decline in family functioning in areas such as problem-solving, communication, and behavior control, warrants further attention due to the ongoing demographic shift towards an aging population [51]. Underprivileged groups, including Hispanic and non-Hispanic elderly individuals and those with comorbidities demonstrated a stronger preference for ICT use as it helped to bridge the gap in accessing and utilizing relevant services [36].

#### 3.3.3 Attitudinal and practical changes

Seven of the selected articles revealed that elderly individuals experienced both attitudinal and practical changes after using ICT [37,47,54,58,59,61,67]. For instance, Scheibe et al. [59] found that elderly individuals considered a home-based telemonitoring app easy to use, made them feel secure, and they appreciated its regular monitoring of vital parameters. Participants viewed the app as simplifying their daily lives and increasing independence through telemonitoring. Similarly, HIV patients preferred telehealth visits for some or all appointments due to reduced logistics, time consumption, and a more comfortable feeling at home compared to clinics [54]. They also indicated that video telehealth made them perceive that healthcare providers gave them full attention.

Furthermore, participants who had prior experience with the Internet or video/voice/conference calls were more likely to access ICT and preferred replacing in-person appointments with online calls with their usual providers [37]. In addressing elderly ICT use problems, Hawley et al. [67] pre-visited the participants’ homes to set up the devices or guide family members for participants who did not have access. Additionally, they called participants before the study started to discuss how to adjust their audio and video devices, resulting in greater interest in home telehealth visits, acknowledgement of benefits to health and well-being, increased confidence in ICT effectiveness, and willingness to recommend telehealth to friends.

Moreover, participants with negative pre-usage attitudes towards wearable devices described them as useful post-usage, showed interest in learning more, and continued independent use [61]. Regarding mental well-being, elderly individuals reported improved well-being and decreased distress after cognitive training programs/software, recognizing that engagement with new technologies could potentially mitigate negative outcomes from stressful pandemic events and mitigate the effect of hyperarousal symptoms [47]. Finally, the effectiveness of ICT was highly appraised, with all interventions being completed in one clinic visit, averaging three interventions on the same day [58], and patients receiving telemedicine indicating satisfaction and quality-of-life improvements as well as better health status.

## 4 Discussion

This comprehensive review integrated findings from 34 relevant studies and provides valuable insights on understanding ICT adoption among the elderly population during COVID-19. This study expanded the focus of ICT to the healthcare context, emphasizing the possibility of combining innovative ICT to address the healthcare needs of older individuals, going beyond traditional approaches of utilizing ICT for promoting social interaction and mental states [23,70,71]. Three core functions of ICT in the healthcare context were classified: general medical services, personalized care services, and informational provision. This study identified the differences between ICT functions to meet the elderly's varied needs instead of listing technologies embedded into elderly healthcare [72]. The proposed classification of ICT indicates the possibility of leveraging any one of the functionalities to integrate with diverse service modalities. For example, home care and community care were expanded in scale due to the feasibility of delivering online medical services and personalized care services to meet the elderly's demands. With family and community support, even less-literate elders were able to accept new technologies effortlessly [70,73]. Thus, the applications of ICT in healthcare hold potential as a comprehensive facilitator for enhancing the quality of life among the aging population. The role of ICT extends beyond the traditional trend of preventing hospitalization or institutionalization but aims towards the ultimate goal of promoting healthy and active aging [74,75].

This study also offered a comprehensive understanding of the barriers the elderly face at individual, mental, social, and technical levels. While most previous studies only focused on the exploration or discussion of either one or two types of barriers to ICT use among the aging population in the healthcare domain [16,23,76], we suggested a more holistic approach targeting all levels of barriers to maximize ICT adoption and utilization among the elderly. In our study, the most frequently cited barriers were individual weaknesses (e.g., diminishing interest in utilizing ICT, lower socioeconomic status, lack of digital literacy) and technical barriers such as inaccessibility to the Internet, which aligned with previous research that access to computers, degree of support, education level, and other factors impacted the elderly's perception towards ICT, leading to selective use [77]. It is worth noting that social barriers such as limited resources for access have emerged as a critical issue in delivering healthcare services. Elderly individuals residing in rural areas or coming from disadvantaged backgrounds deserve more attention to ensure equitable access to ICT, as they often encounter greater challenges in technical, economic, and social aspects [78]. Additionally, support from close acquaintances would benefit the elderly in overcoming these barriers since trust plays a vital role when trying new technologies, seeking help, and making decisions [[79], [80], [81]]. Healthcare providers should prioritize addressing the identified barriers, as they are associated with diminishing interest in ICT utilization and willingness to try such technologies [82].

Furthermore, our study observed noteworthy changes in the elders’ attitudes and behaviors following their interaction with ICT. Subsequently, improvements were observed in their physical performance, health condition, and psychological well-being as a result of their interaction with these technologies. We observed a shift in attitudes, with increased satisfaction during or after ICT interactions and a greater inclination towards future utilization. This was due to the potential of ICT to enhance the quality of life, maintain health, foster independence, and provide caregiving support, consequently promoting active aging and sustained engagement [83]. Moreover, older individuals with high ICT self-efficacy achieved better social and information interaction through ICT, enabling them to build and strengthen their social network while alleviating loneliness [70].

Though ICT has been debated for being unable to fully substitute offline healthcare in our included studies, and we recognize that current health-related ICT is not yet widely adopted by the elderly, an optimal approach combining the advantages of online and offline healthcare deserves more exploration. The COVID-19 pandemic has facilitated the integration mode with the volume of online medical consultations drastically increasing [84]. Among selected articles, studies indicated the inconvenience of hospitalization and transportation due to COVID-19 restriction policies. Such restrictions provided a natural condition to innovate ICT functions and embed traditional healthcare services. To enhance the utilization of ICT among the elderly population, several theoretical and practical suggestions can be adopted. Firstly, research should prioritize understanding the specific needs and barriers faced by the elderly regarding ICT adoption in healthcare to guide the development of user-friendly and age-appropriate ICT solutions. Secondly, the decision regarding the level of integration level between online and offline services should be tailored to the unique national conditions, taking into account factors such as the extent of population aging, cultural preferences, specialized developmental modes, cost-effectiveness, and other relevant considerations. Thirdly, the evaluation of effectiveness in adopting ICT should be considered from a more holistic perspective rather than focusing on health outcomes, physical performance, or psychological health. Additionally, there are unresolved ethical and legal concerns that need to be addressed [72]. It is crucial to ensure that the ICT application effectively addresses these concerns as they directly impact the level of disclosure, and consequently, the effectiveness of healthcare service delivery.

This study has provided a systematic review of existing research on the use of ICT by the elderly during the COVID-19 pandemic. However, some limitations should be noted. Firstly, this review focused solely on the elderly, resulting in a modest selection of 34 articles based on the inclusion and exclusion criteria. Meanwhile, excluding pre-COVID-19 papers may limit the understanding of ICT development trends. Future studies should consider including papers from both before and after the COVID-19 period to capture changes in ICT utilization following the public health crisis. Secondly, the included studies mainly relied on qualitative data, thereby constraining the feasibility of conducting a meta-analysis to ascertain the effectiveness of adopted ICT in different countries. To gain more comprehensive statistical insights, future research should collect quantitative data during the data collection phase to enable a comprehensive cross-country meta-analysis to assess ICT adoption among the elderly of different ages, cultures, and backgrounds, which would facilitate a deeper understanding of the trends and variations in ICT development across different countries or cultures. Overall, this review acknowledges the typical limitations associated with systematic reviews (e.g., exclusion of unpublished studies and availability of data). Further investigation into the utilization of health-related ICT among the elderly is necessary to address these limitations by attempting more diversified research methodologies and representative samples.

## 5 Conclusion

This article has presented findings from a systematic literature review of 34 studies examining ICT adoption among the elderly during the COVID-19 pandemic. The research categorized ICT functions and noted barriers as well as attitudinal and practical changes in the elderly using health-related ICT. Despite rapid increases in ICT usage throughout the pandemic, findings indicate a continued need for more comprehensive considerations while designing ICT and delivering healthcare services to support the elderly's health and independent aging in the post-pandemic world. This review contributes insights into the elderly’ subjective experiences with and perspectives on ICT use while informing future ICT development efforts targeting this population.

## Ethics statement

Review and approval by an ethics committee were not needed for this study because this was a systematic review and no human, animal, or clinical risks were involved. For the same reason, informed consent was not required.

## Funding disclosure

This review has no funding affiliation to declare.

## Data availability statement

Data will be made available on request. Data generated and utilized for analyses of results presented in the manuscript are available from the first author on reasonable request, due to the limitations of the review software Rayyan, authors are required to provide the requester's email address to facilitate access to the data.

## CRediT authorship contribution statement

**Sihui Chen:** Writing – review & editing, Writing – original draft, Visualization, Validation, Project administration, Methodology, Investigation, Formal analysis, Data curation, Conceptualization. **Mengyuan Niu:** Writing – review & editing, Writing – original draft, Visualization, Validation, Project administration, Methodology, Investigation, Formal analysis, Data curation, Conceptualization. **Cindy Sing Bik Ngai:** Writing – review & editing, Supervision, Project administration, Methodology, Investigation, Funding acquisition.

## Declaration of competing interest

The authors declare that they have no known competing financial interests or personal relationships that could have appeared to influence the work reported in this paper.
