# Supplementary material for: What is the next step of ICT development? The changes of ICT use in promoting elderly healthcare access: A systematic literature review
Source: Heliyon. 2024 Feb 7;10(3):e25197. doi: 10.1016/j.heliyon.2024.e25197 (PMC10873661; doi:10.1016/j.heliyon.2024.e25197)
Supplement: Multimedia component 1 [file mmc1.doc]

**Appendix A.** Characteristics of included articles

| **Titles** | **Author(s) and publication year** | **Participants characteristics** | **Countries/region of study** | **Functions of ICT** | **Barriers in using ICT** | **Change(s) after using ICT** |
| --- | --- | --- | --- | --- | --- | --- |
| (Vi)-Rushed Into Online Group Schema Therapy Based Day-Treatment For Older Adults By The Covid-19 Outbreak In The Netherlands | Van Dijk et al., 2020 | 64-70 years old patients suffering from chronic and/or treatment-resistant affective disorders and comorbid personality problems. | Netherlands | **Personalized care provision:** Online therapy through video/phone class/email. | N. A | **Attitudinal**: Patients felt being taken seriously because the online group sessions provided them with detailed comments on their homework and brief weekly individual contact with one of the therapists. |
| A Technology Training Program To Alleviate Social Isolation And Loneliness Among Homebound Older Adults: A Community Case Study | Jimenez et al., 2021 | Elderly in general. | United States | **Information provision:** Use computer devices and the Internet to socially connect with new and existing contacts via 1.5–2 hours modules. | **Social barriers**: The intensity of staff time and resources; inadequate participants and volunteer recruitment; challenges in coordinating data sharing efforts across multiple project partners. | N. A |
| Acceptability Of The Voice Your Values, An Advance Care Planning Intervention In Persons Living With Mild Dementia Using Videoconferencing Technology | Vellani et al., 2022 | Older adults with mild dementia. | Canada | **General medical service provision:** Voice Your Values (VYV), a tailored ACP video intervention for persons living with mild dementia and their trusted individuals. | **Social barrier:** Need help from trusted individuals for setting up the network.  **Technical barrier:** Unstable network in rural areas; non-verbal expression on the screen. | **Attitudinal**: All participants rated VYV intervention as acceptable. |
| Accessibility And Utilisation Of Telehealth Services Among Older Adults During Covid-19 Pandemic In The United States | Ng et al., 2022 | Community-dwelling aged over 65 years old. | United States | **General medical service provision:** Telehealth. | **Social barrier:** Living in a non-metro area.   **Technical barrier:** Having no access to the Internet. | **Practical**: Hispanic, non-Hispanic Black and those with comorbidities incline to use telehealth services due to shorten disparity in accessing and using telehealth. |
| Accessibility Of Telehealth Services During The Covid-19 Pandemic: A Cross-Sectional Survey Of Medicare Beneficiaries | Ng and Park, 2021 | Elderly aged over 65 years old. | United States | **General medical service provision:** Telehealth. | **Individual weakness:** Increased age; lower income; lack of experience in participating in video, voice, or conference calls over the Internet.  **Technical barrier:** The accessibility of the Internet and technology devices. | **Attitudinal**: Beneficiaries who had prior experiences were more inclined to report having access to telehealth. **Practical**: Non-Hispanic Black beneficiaries and those with comorbid conditions would use telehealth as an alternative to replacing a regularly scheduled in-person appointment. |
| Addressing Covid-19 Challenges In A Randomised Controlled Trial On Exercise Interventions In A High-Risk Population | Kienle et al., 2021 | Elderly with a risk of falling. | Germany | **Personalized care provision:** Telemedicine such as video and phone calls. | **Individual limitation:** limited functioning, telemedical experiences, and experiences in handling technique tools. **Social barrier:** Inequity in using smart electronic devices. | N. A |
| Assessing The Factors That Influence The Adoption Of Healthcare Wearables By The Older Population Using An Extended Pmt Model | Singh et al., 2022 | Elderly aged over 60; living in Delhi and the national capital region; earning less than 200,000 INR. | India | **Personalized care provision:** Healthcare wearable devices (hwds). | **Individual weakness:** hwds are more attracted to the younger elderly. | N. A |
| Baduanjin Qigong Intervention By Telerehabilitation (Teleparkinson): A Proof-Of-Concept Study In Parkinson's Disease | Carvalho et al., 2021 | Elderly aged around 75 years old, with Parkinson's disease in Canada. | India | **Personalized care provision:** Telerehabilitation. | **Technical barrier:** Technical issues were encountered, and the participants had difficulty understanding the procedure the first time. | **Practical**: Participants scored better in the overall quality of life after the intervention, especially with tasks related to daily living and mobility; their walking performance has been improved such as self-selected and fast-paced gait speed as well, static and dynamic balance. |
| Barriers To Conducting Deprescribing In The Elderly Population Amid The Covid-19 Pandemic | Elbeddini et al., 2021 | N. A | Canada | **Personalized care provision**: Virtual care delivers deprescribing services and electronic health records, which consist of the complete record of a patient's health history. | **Individual weakness:** Inability to use technology; lack of literacy; insufficient income to purchase access devices.  **Social barrier:** Lack of resources (e.g., video camera, smartphone) and assistance from others; greater propensity for withdrawal effects; and greater risk for hospitalization. | N.a. |
| Barriers To Learning A New Technology To Go Online Among Older Adults During The Covid-19 Pandemic | Li et al., 2021 | Community-dwelling elderly aged over 65 years old. | United States | **General medical service provision:** Contact health providers virtually.  **Information provision:** Getting more information through smartphone, computer, ipad or online Apps; using for shopping; contacting family/friends virtually; conducting online activities. | **Individual weakness:** no experience in learning new technology to go online; increasing age; below high school education; reduced income; self-reported fair or poor general health. | **Practical**: There is an increase in using ICT during the COVID-19 pandemic. |
| Challenges And Opportunities Of Telehealth Digital Equity To Manage Hiv And Comorbidities For Older Persons Living With Hiv In New York State | Baim-Lance et al., 2022 | Elderly over aged 50 years old, with HIV, and an average of 4 comorbidities. | United States | **Personalized care provision:** Telehealth. | **Individual weakness:** Participants with less formal education reported telehealth visit is worse than in-person visit by describing it as less interpersonal and resulting in poorer outcomes.  **Technical barrier:** Technology was not only a barrier to access, but also influenced perceptions of quality; access is a main problem, especially in video telehealth over phone telehealth. | **Attitudinal**: Participants who did not have access and use barriers preferred telehealth visits some or all the time, they felt more comfortable with taking a medical visit at home compared to clinics. **Practical**: Telehealth visits were easier in logistics, less time-consuming, minimized in-office wait times, and have the full attention of healthcare providers. |
| Community Engagement In The Telehealth Service For Aged People With Diabetes: Covid-19 Response In Bangladesh | Islam et al., 2021 | Elderly aged over 60. | Bangladesh | **Personalized care provision:** Telehealth. | **Individual weakness:** Participants with less formal education reported telehealth visit is worse than in-person visit by describing it as less interpersonal and resulting in poorer outcomes.  **Technical barrier:** Technology was not only a barrier to access, but also influenced perceptions of quality; access is a main problem, especially in video telehealth over phone telehealth. | **Attitudinal**: Participants who did not have access and use barriers preferred telehealth visits some or all the time, they felt more comfortable with taking a medical visit at home compared to clinics. **Practical**: Telehealth visits were easier in logistics, less time-consuming, minimized in-office wait times, and have the full attention of healthcare providers. |
| Dementia Caregiver Virtual Support-An Implementation Evaluation Of Two Pragmatic Models During Covid-19 | Weems et al., 2021 | Older adults with Alzheimer's and related dementias. | United States | **General medical service provision:** Virtual sessions. | **Technical barrier:** Problems with virtual programming, such as tech issues and distractions during delivery. | **Attitudinal**: Appreciated the strength and help of group facilitators and reported enhanced connectedness related to virtual support. |
| Disparities In Telemedicine Use For Subspecialty Diabetes Care During Covid-19 Shelter-In-Place Orders | Haynes et al., 2021 | Elderly patients from one medical center. | United States | **Personalized care provision:** Telemedicine visit for diabetes care. | **Individual weakness:** Increased age and language barrier.  **Mental burden:** Perceive telemedicine as low-quality care.  **Social barrier:** Lack of medical insurance to reimburse a high cost in telemedicine; perceived quality of care. | N. A |
| E-Mail-Based Health Care In Patients With Dementia During The Pandemic | Altunkalem Seydi et al., 2022 | Elderly over aged 70 years old. | Turkey | **Personalized care provision:** Telemedicine through email. | **Individual Weakness:** The inability to perform a physical examination and lack of digital literacy.  **Social barrier:** Lack of eye contact and face-to-face interaction might not be objective enough to evaluate physical function. | **Practical**: Patients who emailed had their existing medical problems resolved before coming to the hospital and avoided peak times; telehealth prevents unnecessary admissions and economic burden by offering home treatment, non-pharmacological recommendations, and referring patients to relevant specialists or emergencies accordingly and effectively. |
| Independent Use Of A Home-Based Telemonitoring App By Older Patients With Multimorbidity And Mild Cognitive Impairment: Qualitative Study | Scheibe et al., 2021 | Older patients with multimorbidity and mild cognitive impairment (MCI) at an average of 78.7 years old. | Germany | **Personalized care provision:** Home-based telemonitoring app. | **Individual weakness:** Short-term memory.  **Mental burden:** anxiety with using the app. | **Attitudinal**: All patients reported the telemonitoring app is easy to use; they have an increased feeling of security and appreciated regular monitoring of vital parameters. **Practical**: patients were getting more independent due to telemonitoring, and the app has simplified their everyday life. |
| Older Adults With Functional Limitations And Their Use Of Telehealth And During Covid-19 | Kim and Ang, | Elderly over 65 years old. | United States | **General medical service provision:** Access to a computer; use of the Internet; communicate with usual health provider by video calls. | **Individual weakness:** Individual functional limitations. | N. A |
| Parkinson’s Patients Situation During The Sars Cov-2 Pandemic And Their Interest In Telemedicine A Cross-Sectional Study | Witt et al., 2021 | Parkinson patients. | Germany | **Personalized care provision:** video therapy and consultation. | **Individual weaknes**s: lack of or not having experience in using ICT; could not imagine video therapy and medical video consultation. | **Attitudinal**: Welcomed technical support for the implementation of video therapy. |
| People Aging With Hiv—Protecting A Population Vulnerable To Effects Of Covid-19 And Its Control Measures | Schmalzle et al., 2022 | HIV patients age over 65 years old. | United States | **General medical service provision:** Video call for mental and behavioral health support.  **Personalized care provision:** (1) Telemedicine: Pharmacists conducted telephonic medication reconciliations for patients; (2) Assisted Service: phone calls to ask about daily-life needs.  **Information provision:** Learning vaccination-related information. | **Mental burden:** Patients are hesitant in receiving vaccinations if getting suggestions through telehealth.  **Social barriers:** Misinformation between clinical and non-clinical staff.  **Technical barriers:** Technological barriers to telehealth appointments. | **Practical**: This shift towards telemedicine decongested traditional clinic space and allow clinicians a greater space for running the run a COVID-19 assessment center. |
| Perspectives Of Older Women With Early Breast Cancer On Telemedicine During Post-Primary Treatment | Buse et al., 2022 | Elderly with an average of age 74 years old, between high school to an advanced degree, post-primary treatment for stage I–III breast cancer, in North Carolina. | United States | **Personalized care provision:** Telemedicine such as video and phone calls. | **Social barrier:** Virtual care lacks personal connection and physical exams, which are important to patients and vary depending on their illness stage.  **Technical barrier:** Some patients opt for phone calls due to technical difficulties accessing the video. | **Attitudinal**: Patients showed a preference for video telehealth as it allows them to convey expressive cues through body language, making it feel more "personal" while enabling clinicians to exhibit a higher level of care, gain more insight and build stronger connections. |
| Rapid Integration Of Home Telehealth Visits Amidst Covid-19: What Do Older Adults Need To Succeed? | Hawley et al., 2020 | Veterans who attended a geriatric renal clinic visit, either in person or through video. | United States | **Personalized care provision:** Video telehealth occurred in patient's homes. | **Mental burden:** Lack of confidence in participation.  **Technical barrier:** Access to technology (i.e., Have an access device, but do not have internet). | **Attitudinal**: Participants reported increased interest and confidence and would recommend ICT to their friends. **Practical**: Internet accessibility, devices, or connection problems have been solved prior to the telehealth start by making a pre-visit which saved the participant's commute time, digital inefficiency, and improved health and well-being. |
| Rapid Transition To Telehealth Group Exercise And Functional Assessments In Response To Covid-19 | Jennings et al., 2020 | Elderly over 65 years old. | United States | **Personalized care provision:** Virtual medical care through a virtual platform. | N. A | N. A |
| Remaining Agile In The Covid-19 Pandemic Healthcare Landscape – How We Adopted A Hybrid Telemedicine Geriatric Oncology Care Model In An Academic Tertiary Cancer Center | Chen et al., 2022 | Elderly with cancer aged over 65 years old. | Singapore | **Personalized care provision:** Telemedicine over the telephone, text messages, and video/audio platforms. | **Individual weakness:** Impaired hearing and vision, cognitive impairment, limited digital literacy. | **Attitudinal**: Participants were satisfied with the telemedicine mode and reported improved quality of life and health status. **Practical**: The average time spent in the hospital per visit was reduced from four hours to two and one-half hours and related interventions were completed in a one-stop clinic with an average of 3 interventions on the same day. |
| Remote Neuropsychological Evaluation Of Older Adults | Tsiakiri et al., 2022 | Participants with an average of 75 years old, were categorized as a) Mild Cognitive Impairment (MCI), (b) Alzheimer’s disease, and (c) cognitively healthy older adults in Greece. | Greece | **General medical service provision:** Telemedicine for early diagnosis and video teleconference for neuropsychological assessment. | **Mental burden:** Elders might feel anxious when they face new technology; concerned with privacy issues associated with transforming sensitive information.  **Social barrier:** Less attention to necessary technological equipment (adequately high internet speed, good camera quality, functioning microphone). | **Practical**: Proved the efficiency of using video teleconference to test patients' neuropsychology; the results of the tests were measured as accurately as with the traditional paper and pencil method. |
| Remotely Administered Resilience- And Wisdom-Focused Intervention To Reduce Perceived Stress And Loneliness: Pilot Controlled Clinical Trial In Older Adults | Jeste et al., 2023 | Elderly over 65 years old. | United States | **General medical service provision:** Use teleconference for conducting psychological training courses. | N. A | **Practical**: Reduction in perceived stress and loneliness, and increased in resilience, happiness, and components of wisdom and positive perceptions of aging. |
| Smartphone-Based Healthcare Apps For Older Adults In The Covid-19 Era: Heuristic Evaluation | Saeidnia et al., 2022 | Elderly in general. | Canada and Australia | **Personalized care provision:** General home-based telemonitoring app. | **Technical barrier:** Interface design is not friendly; the absence of a standardized tool or questionnaire (e.g., SUS) for review of user interface design apps aimed at older adults. | N. A |
| Technology Enhanced Health And Social Care For Vulnerable People During The Covid-19 Outbreak | Romanopoulou et al., 2021 | Elderly over 60 years old and have suffered from at least one health issue. | Greece | **Genera medical service provision:** A cognitive training programme/software. | **Mental burden:** The software was perceived as marginally acceptable by participants. | Attitudinal: Participants showed improved well-being and tended to report decreased subjective distress caused by COVID-19. Practical: Engagement with new technologies can potentially minimize the negative outcomes occurred in stressful situations and mitigate the effect of hyperarousal symptoms. |
| Technology Learning And The Adoption Of Telehealth Among Community-Dwelling Older Adults During The Covid-19 Outbreak | Qin, 2022 | Elderly aged over 70 years old. | United States | **General medical service provision:** Emails and video calls to communicate with healthcare providers. | N. A | **Practical**: Older adults have substantially increased the utilization of telehealth during the COVID-19 outbreak. |
| Telehealth In A Washington, Dc African American Religious Community At The Onset Of Covid-19: Showcasing A Virtual Health Ministry Project | Shelton et al., 2021 | African American older adults living in religious communities. | United States | **Personalized care provision:** Using Air Call phone or Zoom call to provide appropriate treatment and services.  **Information provision:** Facebook and Instagram are only used to promote outreach programs; the e-mail system was used to coordinate between team members and the churches to provide additional resources. | **Social barrier:** Trust in the religious community; inequity in providing social support resources. | N. A |
| The Effect Of A Tele-Health Intervention Program On Home-Dwelling Persons With Dementia Or Mci And On Their Primary Caregivers During The Stay-At-Home-Order Period In The Covid-19 Pandemic Outbreak: Evidence From Taiwan | Lai et al., 2022 | Elderly individuals receiving long-term care services, were recruited from the dementia care center. | Taiwan | **General medical service provision:** Digitally deliver a one-hour synchronous distance teaching class for various health promotion activities. | N. A | **Practical**: The intervention program significantly increased the well-being of the participants and their primary caregivers, and the loss of family function warrants attention. |
| The Impact Of A Telehealth Intervention On Activity Profiles In Older Adults During The Covid-19 Pandemic: A Pilot Study | Johnson et al., 2021 | Elderly over 65 years, cognitively intact, able to read and speak the English language fluently, able to wear an accelerometer, have a body mass index ≥18.5 kg per meters-squared (i.e., not underweight), and able to engage in physical activity. | United States | **General medical service provision:** Google Classroom to view didactic materials related to practicing healthy lifestyle behaviors (i.e., physical activity). | N. A | **Practical**: Computer time and physical activity participation increased, and transportation time decreased. |
| The Study On Public-Interest Short Message Service (Sms) In China During The Covid-19 Pandemic: Mobile User Survey And Content Analysis | Yu et al., 2021 | Elderly over 60 years old in China. | China | **Information Provision:** Text messaging. | N. A | **Attitudinal**: Participants gave a higher evaluation on content and necessity; read almost every COVID-19 text message carefully. |
| Understanding Older Adults’ Attitudes Toward Mobile And Wearable Technologies To Support Health And Cognition | Fowe and Boot, 2022 | Elderly aged over 60 years old from Florida State University. | United States | **Personalized care provision:** Wearable and mobile technologies. | **Individual weakness:** Poorer self-rated health was related to negative attitudes toward these technologies.  **Mental burden:** Privacy concerns were correlated with the intention to adopt the technologies described. | **Attitudinal**: Participants had more positive than negative attitudes and found that the health-supporting technologies are useful and interested in learning more.  **Practical**: Participants would adopt the technologies by themselves and recommend the technologies to others. |
| Va Video Connect For Clinical Care In Older Adults In A Rural State During The Covid-19 Pandemic: Cross-Sectional Study | Padala et al., 2020 | Older veterans who had appointments at clinics. | United States | **Personalized care provision:** A videoconferencing app, VA Video Connect (VVC), to connect for regular clinical care. | **Technical barrier:** Internet accessibility. | N. A |
